# Applications of UAS in Crop Biomass Monitoring: A Review

**DOI:** 10.3389/fpls.2021.616689

**Published:** 2021-04-09

**Authors:** Tianhai Wang, Yadong Liu, Minghui Wang, Qing Fan, Hongkun Tian, Xi Qiao, Yanzhou Li

**Affiliations:** ^1^College of Mechanical Engineering, Guangxi University, Nanning, China; ^2^College of Civil Engineering and Architecture, Guangxi University, Nanning, China; ^3^Guangdong Laboratory of Lingnan Modern Agriculture, Shenzhen, Genome Analysis Laboratory of the Ministry of Agriculture and Rural Area, Agricultural Genomics Institute at Shenzhen, Chinese Academy of Agricultural Sciences, Shenzhen, China; ^4^Guangzhou Key Laboratory of Agricultural Products Quality & Safety Traceability Information Technology, Zhongkai University of Agriculture and Engineering, Guangzhou, China

**Keywords:** unmanned aerial systems, unmanned aerial vehicle, remote sensing, crop biomass, smart agriculture, precision agriculture

## Abstract

Biomass is an important indicator for evaluating crops. The rapid, accurate and nondestructive monitoring of biomass is the key to smart agriculture and precision agriculture. Traditional detection methods are based on destructive measurements. Although satellite remote sensing, manned airborne equipment, and vehicle-mounted equipment can nondestructively collect measurements, they are limited by low accuracy, poor flexibility, and high cost. As nondestructive remote sensing equipment with high precision, high flexibility, and low-cost, unmanned aerial systems (UAS) have been widely used to monitor crop biomass. In this review, UAS platforms and sensors, biomass indices, and data analysis methods are presented. The improvements of UAS in monitoring crop biomass in recent years are introduced, and multisensor fusion, multi-index fusion, the consideration of features not directly related to monitoring biomass, the adoption of advanced algorithms and the use of low-cost sensors are reviewed to highlight the potential for monitoring crop biomass with UAS. Considering the progress made to solve this type of problem, we also suggest some directions for future research. Furthermore, it is expected that the challenge of UAS promotion will be overcome in the future, which is conducive to the realization of smart agriculture and precision agriculture.

## Introduction

Agriculture plays an important role in maintaining all human activities. By 2050, population and socioeconomic growth are expected to double the current food demand (Niu et al., 2019). To solve the increasingly complex problems in the agricultural production system, the development of smart agriculture and precision agriculture provides important tools for meeting the challenges of sustainable agricultural development (Sharma et al., 2020). Biomass is a basic agronomic parameter in field investigations and is often used to indicate crop growth status, the effectiveness of agricultural management measures and the carbon sequestration ability of crops (Bendig et al., 2015; Li W. et al., 2015). Fast, accurate and nondestructive monitoring of biomass is the key to smart agriculture and precision agriculture (Lu et al., 2019; Yuan et al., 2019).

Traditional biomass measurement methods are based on destructive measurements that require the manual harvesting (Gnyp et al., 2014), weighing and recording of crops, which makes large-scale, long-term measurements challenging and time-consuming, and these measurements are not only time-consuming and laborious but also difficult to apply over large areas (Boschetti et al., 2007; Yang et al., 2017). In other research areas, many studies have used satellite remote sensing to monitor biomass. Navarro et al. (2019) used Sentinel-1 and Sentinel-2 data to monitor the aboveground biomass (AGB) of a mangrove plantation. However, meteorological conditions have a great influence on satellite images, such as cloud and aerosol interference, surface glare and poor synchrony with tides (Tait et al., 2019). In addition, satellite data cannot provide sufficient data resolution for precision agricultural applications (Jiang et al., 2019; Song et al., 2020), and it is difficult to obtain timely and reliable data (Prey and Schmidhalter, 2019). Similar to satellite remote sensing, manned airborne equipment can cover a wide range, but the data are not detailed enough (Sofonia et al., 2019; ten Harkel et al., 2020). Meanwhile, although vehicle-mounted equipment can guarantee high accuracy, it has poor flexibility and slow speed (Selbeck et al., 2010; Tian et al., 2020). Unmanned aerial systems (UAS) represent a noncontact and nondestructive measurement method that can obtain the spectral, structural, and texture features of the target at different spatiotemporal scales (Jiang et al., 2019). These systems have the ability to obtain high spatial and temporal resolution data and have great application potential (Niu et al., 2019; Ramon Saura et al., 2019).

To date, most reviews of UAS in the field of agriculture are general reviews involving multiple fields in agriculture, and the description of biomass monitoring is not detailed enough (Hassler and Baysal-Gurel, 2019; Kim et al., 2019; Maes and Steppe, 2019). Reviews of remote sensing for crop biomass monitoring are rare and mainly introduce satellite remote sensing, while the application of UAS in crop biomass monitoring is rarely introduced (Chao et al., 2019). Therefore, the motivation of our study was to conduct a comprehensive review of almost all UAS-related studies in the field of crop biomass monitoring, including information on the equipment used in the field of crop biomass monitoring, biomass indices, and data processing and analysis methods. Finally, the relevant applications are reviewed according to different development directions.

## The Composition of UAS

Unmanned aerial systems consist of unmanned aerial vehicle (UAV) platforms, autopilot systems, navigation sensors, mechanical steering components, data acquisition sensors, and other components (Jeziorska, 2019), among which the most important are the data acquisition sensors (Toth and Jozkow, 2016). Meanwhile, the type of UAV platforms and flight conditions will have a great impact on the data acquisition process of sensors, which need to be considered (Domingo et al., 2019; ten Harkel et al., 2020).

### UAV Platforms

**FIGURE 1:**
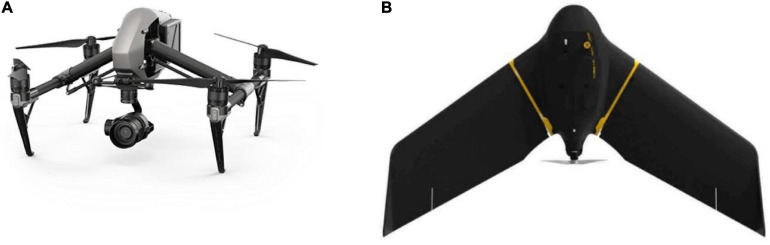
**(A)** DJI Inspire 2 Rotor Drone and **(B)** eBee X Fixed-Wing Drone.

The most commonly used platforms in crop biomass monitoring are fixed-wing drones and rotor drones (Hassler and Baysal-Gurel, 2019). Hogan et al. (2017) summarized the characteristics of fixed-wing aircrafts and rotorcrafts. Fixed-wing aircrafts usually have a larger payload capacity, faster flight speed, longer flight time, and longer range than rotorcrafts. For these reasons, fixed-wing systems are particularly useful for collecting data over large areas. Fixed-wing aircrafts have poor mobility, need more space to land, and have more expensive prices than rotor UAVs. Rotor UAVs are very maneuverable and can hover, rotate and take pictures at almost any angle. Although there are also expensive models, more low-cost models have widely appeared in the market. Compared with fixed-wing aircrafts, the main disadvantage of rotor UAVs is their short range and flight time. Figure 1 shows DJI Inspire 2 Rotor Drone^1^ and eBee X Fixed-Wing Drone^2^.

The flight planning of a fixed-wing UAV is very similar to that of a manned aircraft, while a rotor UAV can meet almost any trajectory requirements, including hover, slow motion and attitude control (Toth and Jozkow, 2016). These features enable rotor UAVs to perform extremely accurate tasks (Kim et al., 2019). Therefore, rotor UAVs are more commonly used in biomass monitoring than fixed-wing aircrafts.

### Data Acquisition Sensors

**FIGURE 2:**
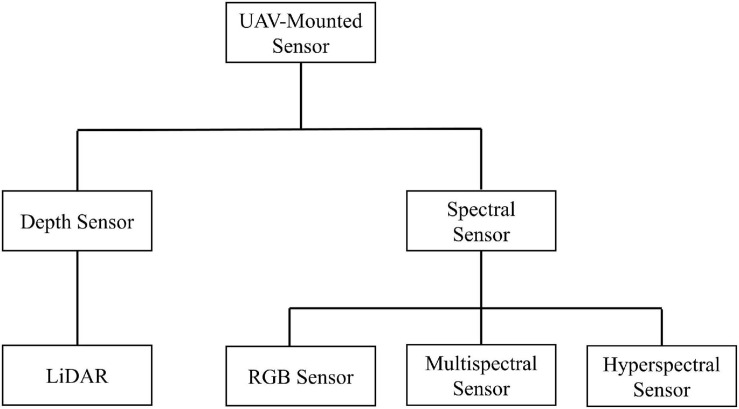
UAV-mounted sensor types.

Unmanned aerial systems usually obtain data through spectral sensors and depth sensors (Toth and Jozkow, 2016). Spectral sensors mainly include RGB sensors, multispectral sensors, and hyperspectral sensors, which can obtain color and texture information from the crop surface (Li et al., 2019). The difference between these three types of sensors is their ability to sense the spectrum (Shentu et al., 2018; Zhong et al., 2018; Kelly et al., 2019). Light detection and ranging (LiDAR) is a typical example of a depth sensor and can clearly obtain the three-dimensional structure and height information of crops (Wijesingha et al., 2019). Figure 2 shows several UAV-mounted sensor types.

#### Spectral Sensors

Based on the same imaging principle, RGB, multispectral, and hyperspectral sensors all capture images by sensing spectral bands but have abilities to sense different spectral bands.

RGB sensors are a type of visible light camera that can detect three bands of color: red (R), green (G), and blue (B) (Shentu et al., 2018). The data from the three bands represent the intensity of R, G, and B in each pixel (Tait et al., 2019). Although RGB sensors have low accuracy compared with other sensors because they can collect spectral data from only three bands, the low-cost characteristic of RGB sensors is not possessed by others. Due to the need for low cost during the large-scale use of UAS in the monitoring of crop biomass, RGB sensors have received increasing attention because of their low-cost characteristics (Calou et al., 2019; Lu et al., 2019; Yue et al., 2019). The combination of RGB data with better biomass indices and advanced algorithms can obtain high accuracy at a low cost (Acorsi et al., 2019; Lu et al., 2019; Yue et al., 2019). Therefore, in the field of crop biomass monitoring by UAS, RGB sensors play an irreplaceable role.

Since spectral information is lost during the process of color image recording, the use of RGB input obviously limits the amount of information to extract from the highlighted area (Kelly et al., 2019). Compared with three-channel RGB imaging, multispectral images contain more imaging bands (Shentu et al., 2018).

Multispectral image data containing several near-infrared (NIR) spectral regions are superior to RGB data (Cen et al., 2019), but the disadvantage is that the cost of multispectral sensors is higher than that of RGB sensors (Costa et al., 2020). Different spectral bands can reflect the characteristics of different plants and can be used to effectively distinguish different crops (Xu et al., 2019). Song and Park (2020) used the RedEdge multispectral camera from MicaSense to analyze the spectral characteristics of aquatic plants and found that waterside plants exhibited the highest reflectivity in the NIR band, while floating plants had high reflectivity in the red-edge band.

Hyperspectral sensors can obtain more abundant spectral information than multispectral sensors (Zhong et al., 2018). Yue et al. (2018) used the UHD 185 Firefly (UHD 185 Firefly, Cubert GmbH, Ulm, Baden-Württemberg, Germany) hyperspectral sensor to collect panchromatic images with radiation records of 1000 × 1000 (1 band) and hyperspectral cubes of 50 × 50 (125 bands), with rich texture and spectral information. The disadvantage is that the cost of hyperspectral sensors is higher than that of multispectral sensors. In addition, the spatial resolution of hyperspectral images is lower than that of ordinary images, which may cause the loss of detail information for small targets. Meanwhile, more spectral information may not be useful in some cases. Tao et al. (2020) used hyperspectral sensors to study the correlation between different vegetation indices (VIs) and red-edge parameters and crop biomass. It was found that using too many spectral features as independent variables will lead to overfitting of the model, so it is necessary to use an appropriate number of spectral features that are highly related to biomass.

How to improve the reliability of spectral data is an unavoidable problem when using spectral sensors to collect data. First, the image resolution will affect the results of AGB monitoring, and the higher the image resolution is, the higher the prediction accuracy (Domingo et al., 2019). Yue et al. (2019) found that using image texture information to estimate the best image resolution for AGB monitoring depends on the size and row spacing of the crop canopy. Second, fisheye lenses may have an advantage over flat lenses. Calou et al. (2019) coupled a 16-megapixel plane lens with a 12-megapixel fisheye lens on a UAV for data collection, and the results showed that the fisheye lens estimation was the most accurate at an altitude of 30 m. Finally, at present, some applications using spectral data are processed without accurate or rough calibration. Guo et al. (2019) proposed a general calibration equation that is suitable for images under clear sky conditions and even under a small amount of clouds. The method needs to be further verified.

Although RGB sensors can only collect spectral data from the R, G, and B bands, the equipment is inexpensive. Although hyperspectral sensors can collect spectral information from many bands, the equipment is expensive. Unless there are special requirements for detailed hyperspectral images and the equipment is inexpensive, a multispectral sensor that balances the richness of spectral bands and equipment costs exhibits the highest cost performance and should be the default imaging choice (Hassler and Baysal-Gurel, 2019; Niu et al., 2019).

#### Light Detection and Ranging

**FIGURE 3:**
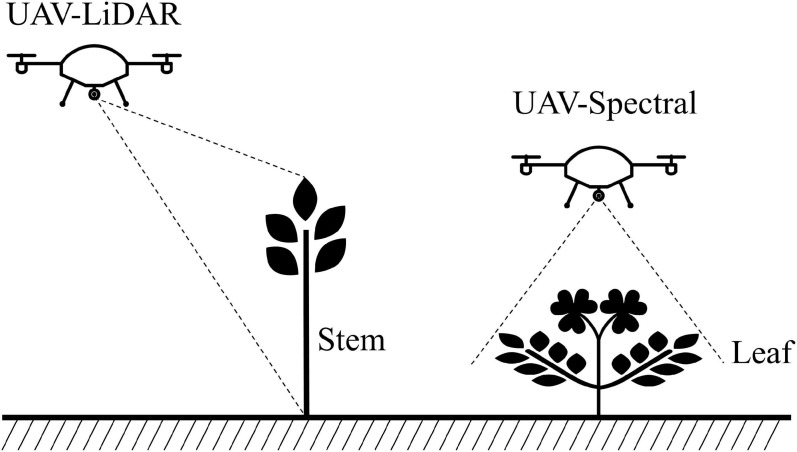
A schematic illustration of the difference between LiDAR and spectral data.

Spectral data have poor robustness in the case of target overlap, occlusion, large illumination changes, shadows, and complex scenes. Depth data that do not change with brightness and color can provide additional useful information for complex scenes (Shuqin et al., 2016). At present, the depth sensors on UAV platforms are mainly LiDAR (Wallace et al., 2012; Qiu et al., 2019; Tian et al., 2019; Wang D.Z. et al., 2019; Yan et al., 2020). LiDAR has become an important information source for the evaluation of the vegetation canopy structure, which is especially suitable for species that limit artificial and destructive sampling (Brede et al., 2017). Figure 3 shows a schematic illustration of the difference between LiDAR and spectral data (Zhu Y.H. et al., 2019).

Light detection and ranging is an active remote sensing technology that accurately measures distance by emitting laser pulses and analyzing the returned energy (Calders et al., 2015). With the development of global positioning system (GPS), inertial measurement unit (IMU), laser and computing technology, which make it possible to use LiDAR more inexpensively and accurately, a LiDAR system based on a UAV platform has become possible (Lohani and Ghosh, 2017). Compared with spectral sensors, LiDAR tends to provide accurate results of biomass prediction (Acorsi et al., 2019) because spectral data tend to be saturated in the middle and high canopy (Féret et al., 2017), and LiDAR can improve this through depth information (Hassler and Baysal-Gurel, 2019).

However, when it is difficult to estimate plant height, it is difficult to accurately monitor biomass through LiDAR. ten Harkel et al. (2020) used a VUX-SYS laser scanner to monitor the biomass of potato, sugar beet, and winter wheat. The researchers achieved good results in monitoring the biomass of sugar beet (*R*^2^ = 0.68, RMSE = 17.47 g/m^2^) and winter wheat (*R*^2^ = 0.82, RMSE = 13.94 g/m^2^), but the reliability for monitoring potato biomass was low. The reason for this result is that potatoes have complex canopy structures and grow on a ridge, and the other two crops have vertical structures and uniform heights. Therefore, for potatoes, it is difficult to visually determine the highest point of a specific position.

Finally, how to improve the data reliability by adjusting the LiDAR parameters is still lacking in more research. For instance, the sampling intensity of LiDAR has an impact on the accuracy of monitoring biomass (Wang D.Z. et al., 2020), but the current research in this area needs to be further verified.

#### Multisensor Fusion

The combination of data obtained from multiple sensors is an effective method to improve the accuracy of biomass estimation. On the one hand, the density of LiDAR point clouds has been improved with increased data resolution and penetrability (Wallace et al., 2012; Yan et al., 2020), which can improve the disadvantage that spectral data collected by RGB, multispectral and hyperspectral sensors are easily saturated in the middle and high canopy (Gitelson, 2004; Féret et al., 2017). On the other hand, the texture and spectral features that can be collected by RGB, multispectral, and hyperspectral sensors are also beyond the reach of LiDAR (Liu et al., 2019; Yue et al., 2019; Zheng et al., 2019). A variety of sensors with different characteristics are used to collect data, and the data that can reflect different characteristics of target crops are combined to provide more effective characteristics that are not cross-correlated that are needed for data analysis with a regression algorithm (Niu et al., 2019) to improve the accuracy of biomass estimation.

Wang et al. (2017) first evaluated the application of the fusion of hyperspectral and LiDAR data in maize biomass estimation. The results show that the fusion of hyperspectral and LiDAR data can provide better estimates of maize biomass than using LiDAR or hyperspectral data alone. Different from the previous methods of using LiDAR and optical remote sensing data to predict AGB separately or in combination, Zhu Y.H. et al. (2019) divided the estimation of maize AGB into two parts: aboveground leaf biomass (AGLB) and aboveground stem biomass (AGSB). AGLB was measuring with multispectral data, which are sensitive to the vegetation canopy. AGSB was measured with LiDAR point cloud data, which are sensitive to the vegetation structure. Compared with using LiDAR data alone or using multispectral data alone, the combination of LiDAR data and multispectral data can more accurately estimate AGB, in which the *R*^2^ increases by 0.13 and 0.30, the RMSE decreases by 22.89 and 54.92 g/m^2^, and the NRMSE decreases by 4.46 and 7.65%.

Other researchers have also carried out many studies in the field of multisensor data fusion and obtained the same results in studies on the monitoring of crop biomass, such as rice (Cen et al., 2019) and soybean (Maimaitijiang et al., 2020). In addition, different crops have different characteristics, and the same crop will show different characteristics under different growth conditions (Johansen et al., 2019; ten Harkel et al., 2020), which requires the use of different sensors to collect crop information comprehensively and screen out some information most related to biomass. The combination of data from multiple sensors is an effective method to improve the accuracy of biomass estimation.

### Flight Parameters

To ensure the most accurate results for biomass monitoring, further tests should be carried out before experiments to determine the optimal flight parameters, such as altitude, speed, location of flight lines, and overlap (Domingo et al., 2019). Increasing the UAV flight height will reduce image resolution (Lu et al., 2019), and the sensitivity of the accuracy of biomass monitoring to the image spatial resolution is an important reference for the configuration of a UAV flight height. The estimation of the other flight parameters did not exhibit much different effects on the overall effect of biomass monitoring from that of standard flight parameters, but different flight parameters can lead to different point densities and distributions, which have a greater impact on biomass monitoring than altitude and velocity. A better crossover model and a closer flight path may improve biomass monitoring overall (ten Harkel et al., 2020). The images need to be overlapped sufficiently to improve the accuracy of biomass monitoring (Borra-Serrano et al., 2019). Therefore, the UAV flight plan should be wide enough. Domingo et al. (2019) found that reducing side overlap from 80 to 70% while maintaining a fixed forward overlap of 90% may be an option to reduce flight time and procurement costs. For specific species, such as rice, due to the physiological characteristics of rice, the analysis of the solar elevation angle during the creation of a flight plan is very important to avoid the influence of sun glint and hotspot effects (Jiang et al., 2019).

## Biomass Indices

It is a common method in biomass monitoring to use biomass indices to obtain data directly related to biomass. Common biomass indices include VIs and crop height (CH), which are extracted from images or three-dimensional point clouds. There are also relevant studies that do not use biomass indices but directly use images or three-dimensional point clouds to conduct correlation analysis with biomass (Nevavuori et al., 2019).

### Vegetation Indices

A variety of VIs from remote sensing images can be used to monitor the state of vegetation on the ground. This method is also able to quantitatively evaluate the richness, greenness and vitality of vegetation. After years of development, VIs can be divided into various monitoring and calculation methods, among which the most commonly used is the normalized difference vegetation index (NDVI) proposed by Rouse et al. (1974). The NDVI is usually used to reflect information such as vegetation cover and growth, and its calculation formula is as follows:

$$N\,D\,V\,I=\frac{N\,I\,R-R}{N\,I\,R+R}$$

Near-infrared is the reflectance in NIR band, and R is the reflectance in red band. The value range of NDVI is (−1, 1). It is generally believed that an NDVI value less than 0 represents no vegetation coverage, while a value greater than 0.1 represents vegetation coverage (Li Z. et al., 2015). Since the index is positively correlated with the density of vegetation, the higher the NDVI value is, the higher the vegetation coverage will be.

Different VIs have unique characteristics, and more spectral features can be identified by using multiple VIs to obtain high monitoring accuracy. Marino and Alvino (2020) used the soil adjusted vegetation index (SAVI), NDVI and OSAVI to characterize 10 winter wheat varieties in a field at different growth stages and obtained optimal biomass monitoring results. Villoslada et al. (2020) combined 13 VIs to obtain the highest accuracy.

Vegetation indices can be built not only on the basis of spectral information but also on the basis of texture information. Texture is an important characteristic for identifying objects or image areas of interest. In several texture algorithms, the gray level co-occurrence matrix (GLCM), which includes variance (VAR), entropy (EN), data range (DR), homogeneity (HOM), second moment (SE), dissimilarity (DIS), contrast (CON), and correlation (COR), which are based on Haralick et al. (1973), is often used to test the effects of texture analysis from UAS data on biomass estimation potential (Zheng et al., 2019).

Vegetation indices based on image texture are usually combined with VIs based on spectral information to monitor crop biomass, and this combination can improve the accuracy of monitoring biomass significantly (Liu et al., 2019; Yue et al., 2019; Zheng et al., 2019). Zheng et al. (2019) predicted rice AGB using stepwise multiple linear regression (SMLR) in combination with VIs and image texture, and the results showed that the combination of texture information and spectral information significantly improved the accuracy of rice biomass estimations compared with the use of spectral information alone (*R*^2^ = 0.78, RMSE = 1.84 t/ha).

Previously, as the required data were obtained by satellites, the spectral data collected would be affected by clouds. When there was cloud cover in the observation area, the information received by the satellite-borne sensor would be all cloud information, instead of reflecting the local vegetation cover (Feng et al., 2009). The low altitude and flexibility of UAS solve this problem, making VIs more widely used. At present, VIs have become indispensable biomass indices for monitoring crop biomass. Common VIs are shown in Table 1.

**TABLE 1:** Introduce the formulation and features of common VIs.

VIs	Formulation	Features	References
Ratio vegetation index	RVI = NIR / R	Monitor the photosynthetically active biomass of plant canopies.	Tucker, 1979
Green chlorophyll index	GCI = (NIR/G) − 1	Estimation of spatially distributed chlorophyll content in crops.	Gitelson et al., 2005
Red-edge chlorophyll index	RECI = (NIR / RE) − 1	Estimation of spatially distributed chlorophyll content in crops.	Gitelson et al., 2005
Normalized difference vegetation index	NDVI = (NIR − R)/(NIR + R)	Quantitative measurement of vegetation conditions over broad regions.	Rouse et al., 1974
Green normalized difference vegetation index	GNDVI = (NIR − G)/(NIR + G)	Nondestructive chlorophyll estimation in leaves.	Gitelson et al., 2003
Green-red vegetation index	GRVI = (G − R) / (G + R)	Monitor the photosynthetically active biomass of plant canopies.	Tucker, 1979
Normalized difference red-edge	NDRE = (NIR − RE) / (NIR + RE)	Increases the sensitivity of NDVI to chlorophyll content by approximately fivefold.	Gitelson and Merzlyak, 1997
Normalized difference red-edge index	NDREI = (RE − G) / (RE + G)	Estimation of senescence rate at maturation stages.	Hassan et al., 2018
Simplified canopy chlorophyll content index	SCCCI = NDRE / NDVI	Real-time detection of nutrient status.	Raper and Varco, 2015
Enhanced vegetation index	EVI = 2.5 × (NIR − R) / (1 + NIR − 2.4 × R)	The EVI remains sensitive to canopy variations while the NDVI is asymptotically saturated in high biomass regions.	Huete et al., 2002
Two-band enhanced vegetation index	EVI2 = 2.5 × (NIR − R) / (NIR + 2.4 × R + 1)	A 2-band EVI (EVI2), without a blue band, which has the best similarity with the 3-band EVI (EVI).	Jiang et al., 2008
Wide dynamic range vegetation index	WDRVI = (a × NIR − R) / (a × NIR + R) (a = 0.12)	The sensitivity of the WDRVI to moderate-to-high LAI (between 2 and 6) was at least three times greater than that of the NDVI.	Gitelson, 2004
Soil adjusted vegetation index	SAVI = (1 + L) (NIR − RE) / (NIR + RE + L)	Almost eliminated soil-induced changes in vegetation index.	Huete, 1988
Optimized soil adjusted vegetation index	OSAVI = (NIR − R) / (NIR − R + 0.16)	Less sensitive to soil background and atmospheric effects.	Rondeaux et al., 1996
Modified chlorophyll absorption in reflectance index	MCARI = [(RE − R) − 0.2 × (RE − G)] × (RE / R)	Evaluate the nutrient variability over large fields quickly.	Daughtry et al., 2000
MCARI/OSAVI	MCARI / OSAVI	Evaluate the nutrient variability over large fields quickly.	Daughtry et al., 2000
Transformed chlorophyll absorption in reflectance index	TCARI = 3 × [(RE − R) − 0.2 × (RE − G) × (RE / R)]	Minimizing LAI (vegetation parameter) influence and underlying soil (background) effects.	Haboudane et al., 2002
TCARI/OSAVI	TCARI / OSAVI	Minimizing LAI (vegetation parameter) influence and underlying soil (background) effects.	Haboudane et al., 2002

*Generally, one or several kinds of VIs should be selected according to different situations. Abbreviate green, red, red-edge, and near-infrared to G, R, RE, and NIR in formulation.*

### Crop Height

Crop height is an important indicator to characterize the vertical structure, and CH is usually strongly correlated with biomass (Scotford and Miller, 2004; Prost and Jeuffroy, 2007; Salas Fernandez et al., 2009; Montes et al., 2011; Hakl et al., 2012; Alheit et al., 2014). The crop surface model (CSM) is an effective CH information extraction technique and has been widely used for different crops (Han et al., 2019).

Crop height data can be obtained using RGB sensors and multispectral sensors. Cen et al. (2019) established a CSM to determine the CH (Tilly et al., 2014) based on spliced RGB images. First, structure from motion (SfM) was used to generate a point cloud, and the specific steps can be found in the study of Tomasi and Kanade (1992). Point clouds consist of matching points between overlapping images such as crop canopies and topographic surfaces. A digital elevation model (DEM) and digital terrain model (DTM) were obtained by classifying the point clouds. The DEM was based on a complete dense point cloud representing the height of the crop canopy, while the DTM was developed from only the surface dense point cloud. The CSM could be obtained by subtracting the DTM from the DEM by importing the two models into ArcGIS software (ArcGIS, Esri Inc., Redlands, CA, United States). Hassan et al. (2019) used a Sequoia 4.0 multispectral camera with the same method to measure the CH of wheat, and the results showed that the correlation between the CH data from UAS and the actual height was very high (*R*^2^ = 0.96).

Crop height data can also be obtained using LiDAR. Zhu W.X. et al. (2019) used CloudCompare open-source software to construct CH raster data from the LiDAR point cloud and studied the effects of CH on monitoring the AGB of crops. The results showed that CH is a robust indicator that can be used to estimate biomass, and the high spatial resolution of the CH data set was helpful to improve the effect of maize AGB estimation.

The monitoring of crop biomass by a single biomass index is sometimes unreliable. On the one hand, it is difficult to obtain reliable CH data from LiDAR in some cases. Johansen et al. (2019) found that dust storms can cause tomato plants to flatten and that once the tomato fruits become large and heavy, the weight may cause the branches to bend downward, thereby reducing the height of the plants. ten Harkel et al. (2020) found that potatoes have complex canopy structures and grow on ridges, so it is difficult to visually determine the highest point of a specific position. In the above cases, VIs can achieve better results than other measurements. On the other hand, CH data can better reflect the three-dimensional information of crops and can more accurately reflect the biomass of crops in the scene of target overlap, occlusion, large changes in light, shadow, and complex scenes. In addition, the information collected by UAS includes not only target crops but also other interference information. If this interference information cannot be effectively eliminated, it will have a negative impact on the monitoring of crop biomass, which can be improved by the combination of multiple biomass indices (Niu et al., 2019). Therefore, the combination of multiple models for biomass estimation is an effective method to improve the accuracy of biomass estimation.

### Multi-index Fusion

The combination of multiple models for biomass estimation is an effective method to improve the accuracy of biomass estimation. The combination of spectral and textural features to construct VIs or the combination of VIs and CH has been shown to improve the results of biomass estimation.

Based on the idea of combining VIs with CH, Cen et al. (2019) used a biomass model that combined VIs and CH to monitor rice biomass under different nitrogen treatments. The results showed that the CH extracted by the CSM exhibited a high correlation with the actual CH. The monitoring model that incorporated RGB and multispectral image data with random forest regression (RFR) significantly improved the prediction results of AGB, in which the RMSEP decreased by 8.33–16.00%, *R*^2^ = 0.90, RMSEP = 0.21 kg/m^2^, and RRMSE = 14.05%.

Relevant studies have proven that a biomass model combined with VIs and CH can also improve the biomass estimation accuracy for corn (Niu et al., 2019), wheat (Lu et al., 2019), ryegrass (Borra-Serrano et al., 2019), and other crops. These cases prove that the combination of VIs and CH is an effective way to build a biomass model. However, Niu et al. (2019) pointed out that the fusion of CH data derived from RGB images in the VIs model, which was based on MLR, did not significantly improve the estimation of the VI model, which may be caused by the clear correlation between VIs and CH in this crop (Schirrmann et al., 2016). Therefore, it is necessary to combine biomass indices reasonably for different crops.

According to the idea of combining spectral information with image texture to build VIs, Liu et al. (2019) used a linear regression model to convert the digital number (DN) of the original image into surface reflectance. The reflectivity obtained from the gain and offset values of each band was used to calculate the VIs and image texture. The results showed that the introduction of image texture into the partial least squares regression (PLSR) and RFR models could estimate winter rape AGB more accurately than a model based on VIs alone. The accuracy of the prediction of AGB by the RFR model using VIs and texture measurements (RMSE = 274.18 kg/ha) was slightly higher than that of the PLSR model (RMSE = 284.09 kg/ha). The same idea has also obtained good results in applications to winter wheat (Yue et al., 2019), rice (Zheng et al., 2019), soybean (Maimaitijiang et al., 2020), and other crops. Biomass models combined with VIs and image texture have great potential in the estimation of crop biomass.

## Data Processing and Analysis Methods

Data analysis is the key link to build the relationship between the data obtained from UAS and the actual crop biomass, and it is an important part of UAS. The data obtained from UAS often contain different noises, and the information is highly correlated. Generally, effective data analysis methods are needed to interpret the data and establish a robust prediction model (Cen et al., 2019). Therefore, scientific and systematic data analysis methods often play an important role.

### Data Preprocessing Methods

Since the data collected by UAS cannot be directly used to monitor biomass, a series of preprocessing steps is needed for the data. When spectral sensors are used, an indispensable step is geometric correction and mosaicking of the image. Common software includes Pix4DMapper and Agisoft Photoscan.

Pix4DMapper software (Pix4D, S.A., Lausanne, Switzerland) is UAS photography geometric correction and mosaic technology based on feature matching and SfM photogrammetry technology. Initially, images were processed in any model space to create three-dimensional point clouds. The point clouds could be transformed into a real-world coordinate system using either direct geolocation techniques to estimate the camera’s location or GCP techniques for automatic identification within the point cloud. The point cloud was then used to generate the DTM required for image correction. Subsequent geographic reference images are linked together to form a mosaic of the study area (Turner et al., 2012).

**FIGURE 4:**
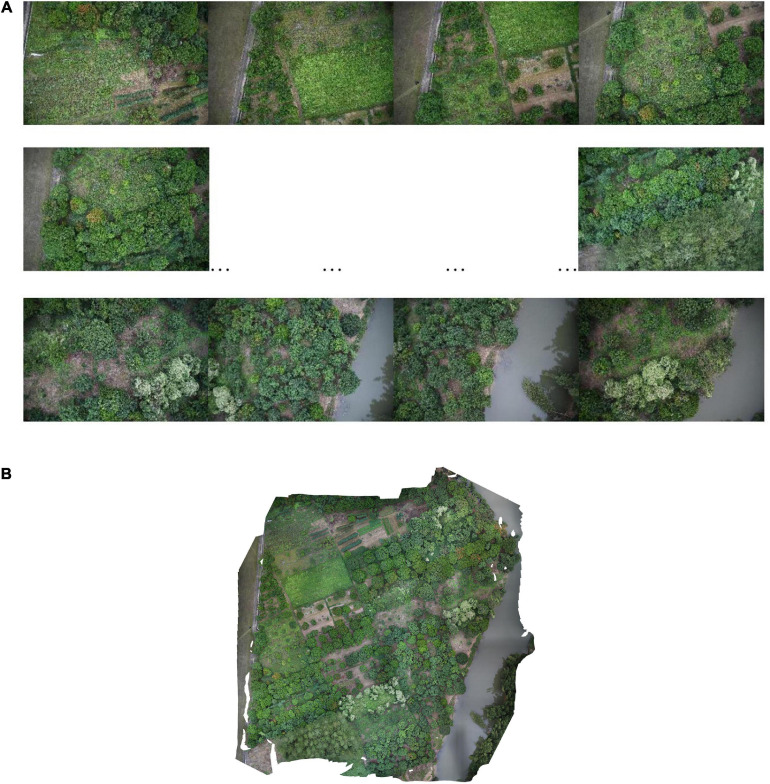
RGB imagery datasets were processed using the software Agisoft PhotoScan. **(A)** High-resolution proof images of the acquisition area. **(B)** Overall map of research area processed by Agisoft PhotoScan.

Agisoft Photoscan software (Agisoft LLC, St. Petersburg, Russia) is also a common UAS data preprocessing software. The processing procedure is similar to that of Pix4DMapper. Finally, the UAS image is exported to TIFF image format for subsequent analysis (Acorsi et al., 2019; Lu et al., 2019). Figure 4 shows RGB imagery datasets were processed using the software Agisoft PhotoScan (Sun et al., 2019).

(a) High-resolution proof images of the acquisition area
(b) Overall map of research area processed by Agisoft PhotoScan.

### Data Analysis Methods

**FIGURE 5:**
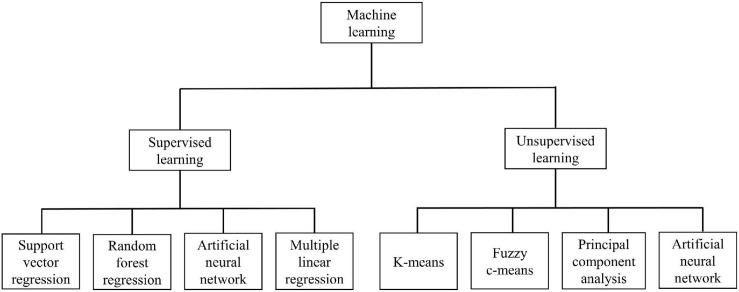
The types of machine learning algorithms.

Machine learning algorithms are widely used to process biomass information. According to whether the input dataset is labeled, machine learning algorithms can be divided into supervised learning algorithms and unsupervised learning algorithms (Dike et al., 2018). Supervised learning algorithms depend on a labeled dataset. Classification algorithms and regression algorithms are the two output forms of supervised learning. In crop biomass monitoring, regression algorithms are more often used than classification algorithms. Because the expected biomass results are often continuous instead of discrete. Unsupervised learning does not rely on a labeled dataset. It is often used when the cost of labeled datasets is unacceptable (Sapkal et al., 2007). This is also a common method to reduce the dimensionality of the data. Most unsupervised learning algorithms are in the form of cluster analysis. Figure 5 shows the types of machine learning algorithms.

Biomass monitoring is a typical regression problem, which can to be solved by supervised learning algorithms. Enough labeled datasets are the basis of supervised learning algorithms. Actual biomass data tend to obtain through destructive samplings (Jiang et al., 2019; Yue et al., 2019). Field trials are limited by the area of cropland and the crop growing season. Therefore, sufficiently large datasets are often not available. How to properly divide the datasets into training datasets and validation datasets is a challenge to train supervised learning algorithms. To solve this problem, Jiang et al. (2019) used fivefold cross validation, Han et al. (2019) used repeated 10-fold cross validation, Zhu W.X. et al. (2019) used leave-one-out-cross validation (LOOCV) to reduce generalization error. Fivefold cross validation and repeated 10-fold cross validation belong to *k*-fold cross validation. LOOCV is a special case of *k*-fold cross validation, in which the number of folds equals the number of instances (Wong, 2015). *k*-fold cross validation divides the datasets into *k* folds, treats each fold as a validation dataset and regards the other *k*−1 folds as a training dataset (Wong and Yang, 2017). The value of folds can be large and the value of replications should be small if *k*-fold cross validation is applied in the classification algorithms (Wong and Yeh, 2020).

#### Support Vector Regression

Support vector regression (SVR) is a boundary detection algorithm for identifying/defining multidimensional boundaries (Sharma et al., 2020), and the basis of this method is to solve the regression problem by using appropriate kernel functions to map the training data to the new hyperspace characteristics and transform the multidimensional regression problem into a linear regression problem (Navarro et al., 2019). Duan et al. (2019) in an analysis of VIs and image texture using SVR found that the SVR itself has the ability to find a suitable combination of different reflectance bands, which shown that SVR has strong adaptability to complex data and is suitable for data analysis in biomass monitoring. Yang et al. (2019) compared PLSR and SVR, and the results showed that the accuracy of SVR was higher than that of PLSR, and the SVR optimized by particle swarm optimization (PSO) could obtain more appropriate parameters and improve the accuracy of the model.

#### Random Forest Regression

Random forest regression is a data analysis and statistical method that is widely used in machine learning and remote sensing research (Viljanen et al., 2018). Compared with artificial neural networks (ANNs), RFR does not suffer from overfitting problems because of the law of large numbers, and the injection of suitable randomness makes them precise regressors (Breiman, 2001). The random forest algorithm makes full use of all input data and can tolerate outliers and noise (Jiang et al., 2019). This algorithm has the advantages of high prediction accuracy, no need for feature selection and insensitivity to overfitting (Tewes and Schellberg, 2018; Viljanen et al., 2018).

#### Artificial Neural Network

An ANN is an information processing paradigm that is inspired by the way biological nervous systems such as the brain process information (Awodele and Jegede, 2013). ANN is a nonparametric nonlinear model that uses a neural network to transmit between layers and simulates reception and processing of information by the human brain (Zha et al., 2020). In individual cases, the results of the algorithm are not better than those of the multiple linear regression (MLR) method. The reason for this difference may be that in these applications, a small sample set will not meet the needs of the artificial neural network (Han et al., 2019; Zhu W.X. et al., 2019; Zha et al., 2020), and compared with RFR, ANN needs large data sets and a large number of repetitions to generate appropriate nonlinear mapping and obtain the optimal neural network (Devia et al., 2019; Han et al., 2019); however, RFR can still be applied for a small amount of sample data (Han et al., 2019; Liu et al., 2019), which leads to more frequent biomass monitoring use of RFR. Therefore, before the development of a deep neural network (DNN), the remote sensing field, including UAS studies, shifted the focus of data analysis methods from ANN to SVR and RFR (Ma et al., 2019).

The appearance of DNN and a series of methods to solve overfitting improved the effect of ANN (Maimaitijiang et al., 2020). Nevavuori et al. (2019) used a convolutional neural network (CNN) to predict the biomass of wheat and barley. The researchers tested the influence of the selection of the training algorithm, the depth of the network, the regularization strategy, the adjustment of super parameters, and other aspects of CNN on the prediction efficiency to improve the monitoring effect. This study proved that if enough information can be collected to increase the number of samples and solve the overfitting problem, ANN will perform no worse than RFR (Zhang et al., 2019).

#### Multiple Regression Techniques

Multiple linear regression (Borra-Serrano et al., 2019; Devia et al., 2019; Han et al., 2019; Zhu W.X. et al., 2019), SMLR (Lu et al., 2019; Zheng et al., 2019) and PLSR (Borra-Serrano et al., 2019; Liu et al., 2019; Yue et al., 2019) are also commonly used multiple regression algorithms. However, with the gradual progress of SVR, RFR, and ANN, these algorithms have gradually become references for SVR, RFR, and ANN and are no longer the main focus of data analysis.

Devia et al. (2019) described an MLR equation for monitoring rice biomass with VIs. In general, there was a linear relationship between the accumulation of biomass and VIs. However, the relationship between biomass and VIs in other crops at maturity can be nonlinear. Therefore, MLR does not apply to these nonlinear relations of crops. Zheng et al. (2019) used SMLR to establish the relationship between rice biomass and remote sensing variables (VIs, image texture, and the combination of VIs and image texture). Although the estimation accuracy was high, the model was complex and difficult to generalize. Moeckel et al. (2018) tested the ability of PLSR, RFR, and SVR to predict the CH of eggplant, tomato and cabbage, and the results showed that PLSR did not exceed the performance of RFR and SVR, so it was excluded first.

The monitoring of biomass is a typical nonlinear problem (Zha et al., 2020). These regression techniques are more suitable for data showing linear or exponential relationships between remote sensing variables and crop parameters (Atzberger et al., 2010; Jibo et al., 2018; Lu et al., 2019). These methods are often not as good as SVR, RFR, and ANN in the monitoring of biomass.

The construction of a high-performance monitoring model based on advanced algorithms (such as machine learning algorithms) is a good method to improve the effect of crop biomass monitoring (Niu et al., 2019). The monitoring of biomass is a typical multi-feature nonlinear problem (Zha et al., 2020), and machine learning algorithms (such as SVR, RFR, and ANN) exhibit superior results in solving these types of problems (Breiman, 2001; Navarro et al., 2019; Maimaitijiang et al., 2020). During the study, by comparing with RFR, the researchers found that ANN was often superior to RFR when dealing with large sample sizes and complex, nonlinear, and redundant data sets (LeCun et al., 2015; Schmidhuber, 2015; Kang and Kang, 2017; Zhang et al., 2018; Maimaitijiang et al., 2020). However, in a small sample size, the lack of samples often leads to the phenomenon of overfitting, and RFR will achieve better results than ANN due to its stronger robustness and generalization ability (Zhang and Li, 2014; Yao et al., 2015; Yuan et al., 2017; Yue et al., 2017; Zheng et al., 2018; Zhu W.X. et al., 2019; Zha et al., 2020).

## The Promotion of Large-Scale UAS Applications

Accuracy is an important indicator to evaluate the effects of UAS in the field of crop biomass estimation. In addition, reducing the cost to promote the large-scale application of UAS in this field is a difficult problem. From the perspective of improving the accuracy of crop biomass estimations, multisensor data fusion, multi-index fusion, the consideration of a variety of features not directly related to the monitoring of biomass, and the use of advanced algorithms are feasible directions (Maimaitijiang et al., 2020). Considering the promotion of large-scale applications, the use of low-cost sensors and the combination of suitable models and algorithms to improve the estimation accuracy of low-cost sensors, rather than the use of more expensive sensors, is an effective research path to promote the large-scale application of UAS in the field of crop biomass monitoring (Acorsi et al., 2019; Lu et al., 2019; Niu et al., 2019; Yue et al., 2019). RGB sensors are currently the most widely used low-cost sensor (Lussem et al., 2019), and studies based on RGB sensors are expected to promote the large-scale application of UAS in the field of crop biomass monitoring.

RGB sensors are not capable of providing NIR band data. Therefore, VIs associated with NIR bands cannot be used, which inhibits the enhancement of vegetation vitality contrast (Lu et al., 2019) and may affect the accuracy of biomass estimation. Lu et al. (2019) used a combination of advanced algorithms and multi-index fusion to compensate for this deficiency. Yue et al. (2019) fused the image texture and VIs to obtain the most accurate estimated value of AGB (*R*^2^ = 0.89, MAE = 0.67 t/ha, RMSE = 0.82 t/ha). These study proves that the use of low-cost sensors can guarantee the accuracy of biomass estimation and is expected to promote large-scale applications.

Solar elevation angle (Jiang et al., 2019), meteorological conditions (Devia et al., 2019; Wang F. et al., 2019), rainfall (Liu et al., 2019; Rose and Kage, 2019), soil characteristics (Acorsi et al., 2019; Vogel et al., 2019), the spatial distribution of multiple plants in a block (Han et al., 2019), and other characteristics not directly related to biomass monitoring also affect the accuracy of biomass estimations. The monitoring accuracy of low-cost sensors can be improved by considering the characteristics that are not directly related to biomass monitoring.

Jiang et al. (2019) calculated the solar elevation angle to avoid sun glint and hotspot effects. In addition, growing degree days (GDD) was incorporated into the model to estimate rice AGB as a meteorological feature. Models incorporating meteorological features achieved better estimation accuracy (*R*^2^ = 0.86, RMSE = 178.37 g/m^2^, MAE = 127.34 g/m^2^) than models that did not use these features (*R*^2^ = 0.64, RMSE = 286.79 g/m^2^, MAE = 236.49 g/m^2^).

Other studies have also demonstrated the importance of considering features that are not directly related to monitoring biomass. Borra-Serrano et al. (2019) also took GDD as a meteorological feature and obtained the best estimate by combining CH, VIs and meteorological data variables in an MLR model (*R*^2^ = 0.81) to monitor ryegrass dry-matter biomass. Devia et al. (2019) also mentioned the influence of solar elevation angle and indicated that weather conditions (sunny and cloudy) can affect the quality of the data, especially in lowland crops where moisture reflection changes the appearance of the image. The above studies showed that the accuracy of biomass estimation can be improved by considering meteorological characteristics and solar elevation angle. More sample points must be obtained from multiple research sites and under different environmental conditions in future studies to train a more robust multivariate model (Liu et al., 2019). Summary of relevant studies are shown in Table 2.

**TABLE 2:** Summarize the equipment, methods, and important results of the studies cited in the body.

Crop	Platforms	Sensors	Biomass indices	Data analysis methods	Results	References
Wheat	DJI Phantom series	A digital camera	VIs, CH	RFR	*R*^2^ = 0.78, RMSE = 1.34 t/ha, RRMSE = 28.98%	Lu et al., 2019
Rice	DJI S1000 DJI Phantom 4 Pro	Mini-MCA 12 multispectral camera DJI FC6310 digital camera	VIs, CH Meteorological feature	SER	*R*^2^ = 0.86, RMSE = 178.37 g/m^2^, MAE = 127.34 g/m^2^	Jiang et al., 2019
Potato Sugar beet Winter wheat	RIEGL RiCOPTER	VUX-SYS laser scanner	CH	MLR	Potato: *R*^2^ = 0.24, RMSE = 22.09 g/m^2^ Sugar beet: *R*^2^ = 0.68, RMSE = 17.47 g/m^2^ Winter wheat: *R*^2^ = 0.82, RMSE = 13.94 g/m^2^	ten Harkel et al., 2020
Maize	DJI Phantom 2	Ricoh GR digital camera	CH	Statistical analysis	The estimated values were most accurate when using a fisheye lens at 30 m altitude.	Calou et al., 2019
Winter wheat	DJI S1000	DSC-QX100 digital camera	VIs	SMLR	*R*^2^ = 0.89, MAE = 0.67 t/ha, RMSE = 0.82 t/ha	Yue et al., 2019
Rice	A lightweight octorotor UAV	An RGB camera A multispectral camera	VIs, CH	RFR	*R*^2^ = 0.90, RMSEP = 0.21 kg/m^2^, RRMSE = 14.05%	Cen et al., 2019
Winter wheat	DJI S1000	DSC–QX100 digital camera UHD 185 Firefly snapshot hyperspectral sensor	VIs	Exponential regression	*R*^2^ = 0.67, MAE = 1.19, RMSE = 1.71	Yue et al., 2018
Winter wheat	DJI S1000	UHD 185-Firefly	VIs	PLSR	The results of AGB monitoring can be improved by combining the red-edge parameters with VIs.	Tao et al., 2020
Corn Wheat	DJI M600 Pro	Mini-MCA 6 multispectral camera	VIs	Linear regression	A systematical radiometric calibration method was proposed.	Guo et al., 2019
Rice	Mikrokopter OktoXL	Tetracam mini-MCA6 multispectral camera	VIs	SMLR	*R*^2^ = 0.78, RMSE = 1.84 t/ha	Zheng et al., 2019
Winter oilseed rape	DJI S1000	Mini-MCA multispectral camera	VIs	PLSR RFR	RFR: RMSE = 274.18 kg/ha PLSR: RMSE = 284.09 kg/ha	Liu et al., 2019
Maize	DJI Phantom 4 Pro DJI M600 Pro	Parrot Sequoia multispectral camera DJI FC6310 digital camera RIEGL VUX-1UAV laser scanner	VIs, CH	MLR PLSR	MLR: *R*^2^ = 0.82, RMSE = 79.80 g/m^2^, NRMSE = 11.12% PLSR: *R*^2^ = 0.86, RMSE = 72.28 g/m^2^, NRMSE = 10.07%	Zhu Y.H. et al., 2019
Soybean	DJI S1000	Mapir Survey2 RGB camera Parrot Sequoia multispectral camera FLIR Vue Pro R 640 thermal imager	VIs, CH	DNN-F2	*R*^2^ = 0.720, RMSE = 478.9 kg/ha, RRMSE = 15.9%	Maimaitijiang et al., 2020
Tomato	DJI Matrice 100	A RGB Zenmuse X3 sensor	VIs	RFR	*R*^2^ = 0.85, RMSE = 0.052 m	Johansen et al., 2019
Ryegrass	Onyxstar HYDRA-12	RGB camera	VIs, CH Meteorological feature	MLR RFR	MLR: *R*^2^ = 0.81, RMSE = 679 kg/ha, NRMSE = 21.3% RFR: *R*^2^ = 0.70, RMSE = 769 kg/ha, NRMSE = 24.2%	Borra-Serrano et al., 2019
Wheat Barley	Airinov Solo 3DR UAV	Parrot’s NIR-capable SEQUIOA-sensor	None	CNN	MAE = 484.3 kg/ha, MAPE = 8.8%	Nevavuori et al., 2019
Ten winter wheat cultivars	Ebee fixed-wing UAV	Canon Powershot S110 RGB camera Canon Powershot S110 NIR camera	VIs	Cluster analysis	Combination of multiple VIs can be a valid strategy.	Marino and Alvino, 2020
Coastal meadows	Ebee fixed-wing UAV	Parrot Sequoia multispectral camera	VIs	RFR	Combination of multiple VIs can be a valid strategy.	Villoslada et al., 2020
Maize	DJI S1000	DSC-QX100 digital camera Parrot Sequoia multispectral camera	BIOVP (VIs, CH)	RFR	*R*^2^ = 0.944, RMSE = 0.495, MAE = 0.355	Han et al., 2019
Bread wheat	DJI Inspires 1 model T600	Sequoia 4.0 multispectral camera	CH	Linear regression	*R*^2^ = 0.96	Hassan et al., 2019
Maize	EWZ-D6 six-rotator UAV DJI M100 four-rotator UAV Ebee fixed-wing UAV	MultiSPEC-4C multispectral camera MicaSense RedEdge-M multispectral camera Alpha Series AL3-32 LiDAR sensor	CH	RFR	*R*^2^ = 0.90, RMSE = 2.29, MRE = 0.22	Zhu W.X. et al., 2019
Rice	An UAV equipped with a Mini-MCA system	An array of 12 individual miniature digital cameras	VIs	SVR	SVR itself has the ability to find a suitable combination of different reflectance bands.	Duan et al., 2019
Winter wheat	Four-axis aerial vehicle UAV 3P	Sony EXMOR HD camera	VIs	SVR	*R*^2^ = 0.9025, RMSE = 0.3287	Yang et al., 2019
Rice	UAV	Tetracam ADC-lite multispectral camera	VIs	MLR	*R*^2^ = 0.76	Devia et al., 2019
Eggplant Tomato Cabbage	DJI 3 Pro	DJI FC300X RGB camera	CH	SVR RFR	*R*^2^ ranging from 0.87 to 0.97 Bias ranging from −0.66 to 0.45 cm	Moeckel et al., 2018
Sorghum	Custom designed UAV platforms	Sony Alpha ILCE-7R Velodyne VLP-16 Two Headwall Photonics push-broom scanners	Four hyperspectral-based features and four LiDAR-based features	PLSR SVR RFR	The data source was more important than the regression method.	Masjedi et al., 2020
Rice	UAV platform	Tetracam ADC-lite multispectral camera	VIs	Multivariable regression	An average correlation of 0.76	Devia et al., 2019

*This table covers plentiful case-studies from different regions for different crops.*

## Conclusion and Future Perspectives

As a high precision, high flexibility and nondestructive remote sensing system, UAS have been widely used to monitor crop biomass. The application of UAS in the monitoring of crop biomass in recent years was reviewed in this article. Four kinds of data acquisition equipment (LiDAR, RGB sensor, multispectral sensor, and hyperspectral sensor), two biomass indices (VIs and CH) and three data analysis methods (SVR, RFR, and ANN) were introduced.

Despite the rapid progress in this area, difficulties remain. First, we need to improve the speed of data acquisition and processing. Although multisensor data fusion improves the accuracy of evaluation, it makes the process of data collection more complex, data sorting more difficult, and objectively reduces the speed of monitoring. In addition, although advanced algorithms improve the evaluation accuracy, they require a long training time. Second, there is no universal method that can be applied to all crops in all cases. Different crops, even the same crops in different environments, have different characteristics. This difference requires us to carefully distinguish the characteristics of crops, use appropriate sensors to collect characteristics, and test multiple indices to determine the best biomass indices. Third, the high cost of equipment hinders the large-scale use of UAS in crop biomass monitoring. Although research on low-cost sensors has appeared, the method that is needed to improve the estimation accuracy when using low-cost sensors still needs further research. It is predicted that adopting multi-index fusion, considering features not directly related to monitoring biomass, and the adoption of advanced algorithms can effectively improve the monitoring effect of low-cost sensors on crop biomass, which is the future development direction.

Because of its high precision, flexibility and nondestructive nature, UAS have the potential to become an important method for the monitoring of crop biomass. Crop biomass monitoring systems based on multisensor fusion and multi-index fusion, the consideration of features that are not directly related to biomass monitoring and the adoption of advanced algorithms are effective methods and development directions to improve the accuracy of crop biomass estimation by UAS. Because of their low cost, using RGB sensors have become an effective method to promote the large-scale application of UAS in the field of crop biomass monitoring. In the field of biomass monitoring, UAS still have great attraction, and there are an increasing number of studies on the monitoring of crop biomass based on UAS. Furthermore, it is expected that the challenges of UAS promotion will be overcome in the future, which is conducive to the realization of smart agriculture and precision agriculture.

## Author Contributions

All authors listed have made a substantial, direct and intellectual contribution to the work, and approved it for publication.

## Conflict of Interest

The authors declare that the research was conducted in the absence of any commercial or financial relationships that could be construed as a potential conflict of interest.

## Footnotes

1 www.dji.com/inspire-2

2 www.sensefly.com/drone/ebee-x-fixed-wing-drone/
